# Supplementary material for: Copy number architectures define treatment-mediated selection of lethal prostate cancer clones
Source: Nat Commun. 2023 Aug 10;14:4823. doi: 10.1038/s41467-023-40315-9 (PMC10415299; doi:10.1038/s41467-023-40315-9)
Supplement: Supplementary file 1 — Supplementary materials [file 41467_2023_40315_MOESM1_ESM.pdf]

Supplementary materials

Supplementary Figures:

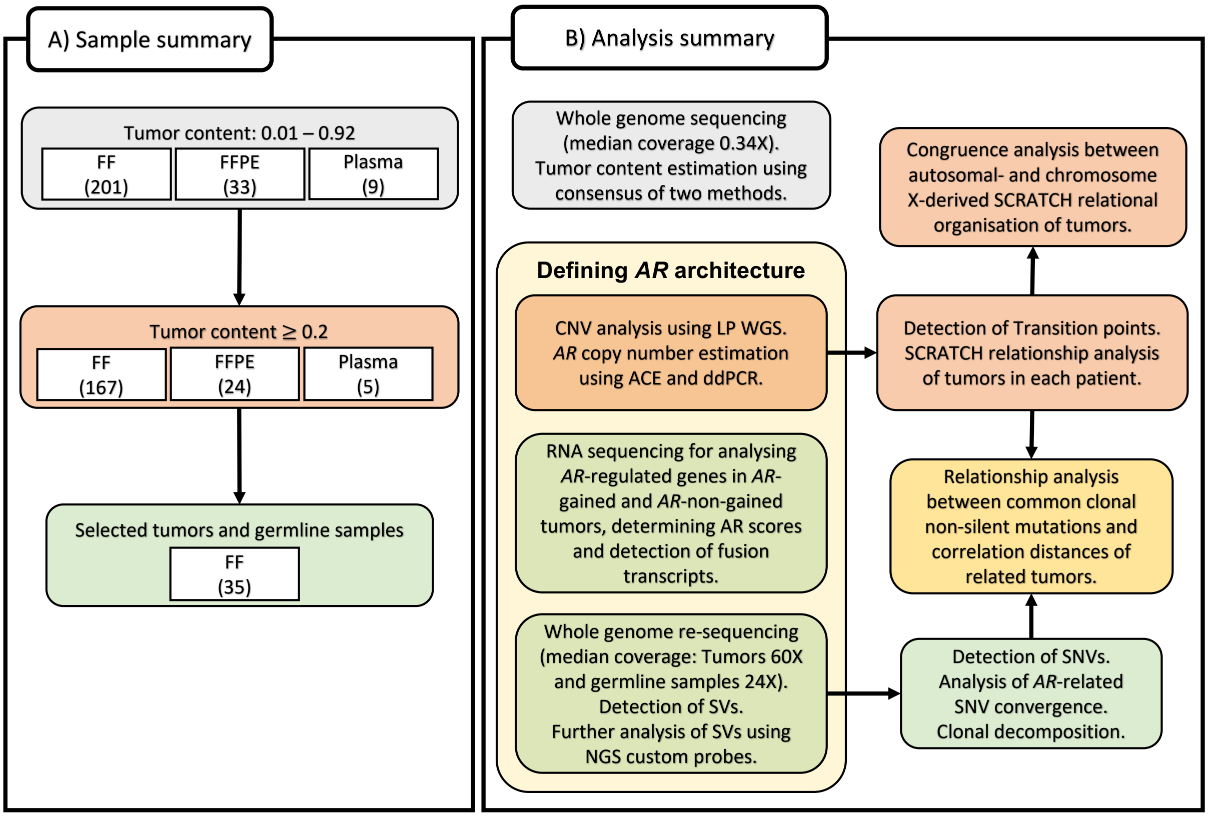

Supplementary Figure 1: Experimental Study Design

a) Sample summary:
(fresh frozen, FF; formalin-fixed paraffin embedded, FFPE; or plasma; sample numbers as shown in parentheses) were subjected to shallow whole genome sequencing (median coverage: 0.34X, range: 0.03-5.88). Samples with estimated tumor content ≥0.2 were selected for further analysis, including *AR* copy number determination and genome-wide copy number assessment. A sub-set of two to six metastases from each patient were chosen as described in the text for re-sequencing at a higher depth of coverage (median coverage: 60X, range 27X-83X). RNA-Seq was performed on 39 samples selected from four patients (CA63, CA76, CA83 and PEA172).
b) Analysis summary:
Copy number analysis on low coverage WGS was performed using ACE package, while somatic structural variants surrounding *AR* gene (chrX:50M-80M) were detected from high coverage WGS data using Delly v-0.7.8. *AR* downstream transcriptional activity was

determined as AR score and, using Arriba, AR fusion transcripts were detected from the transcriptomic data. Furthermore, clonal somatic mutations were detected from 34 tumor cores using Mutect2 and Sclust. On the other hand, relationship among metastases in a patient was determined applying SCRATCH algorithm on copy number transition points. Congruence analysis between autosomal- and chromosome X-based SCRATCH relationship of metastases from two patients (CA63 and PEA172) showed that they are not similar by chance.

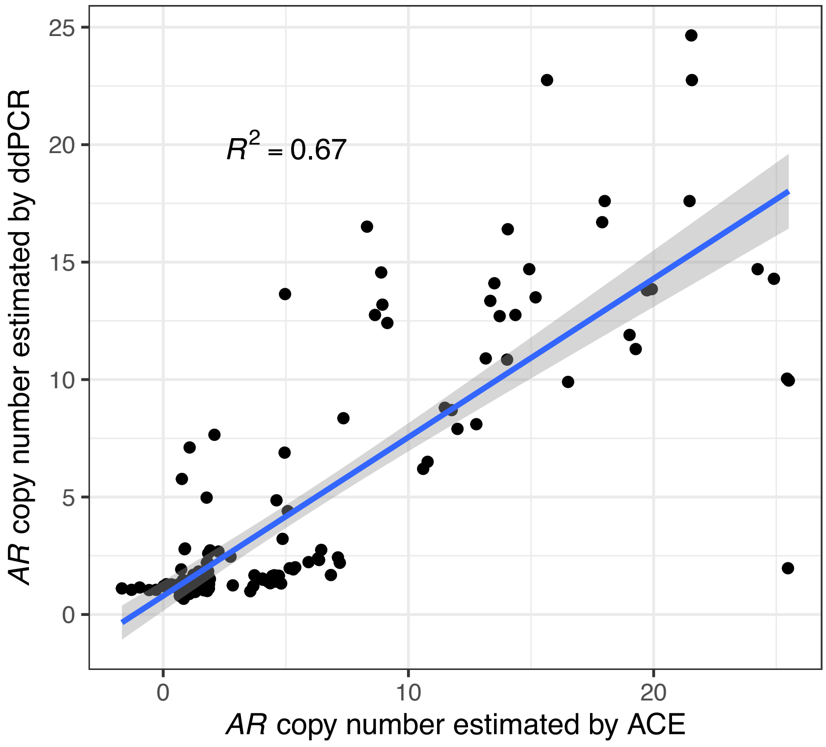

Supplementary Figure 2: *AR* copy number estimation

AR copy numbers for 157 samples estimated by droplet digital PCR (ddPCR, along y-axis) highly correlated with the *AR* copy number estimates from shallow whole genome sequencing (using ACE algorithm, along x-axis). Linear regression line is in blue and confidence interval is shown in grey.

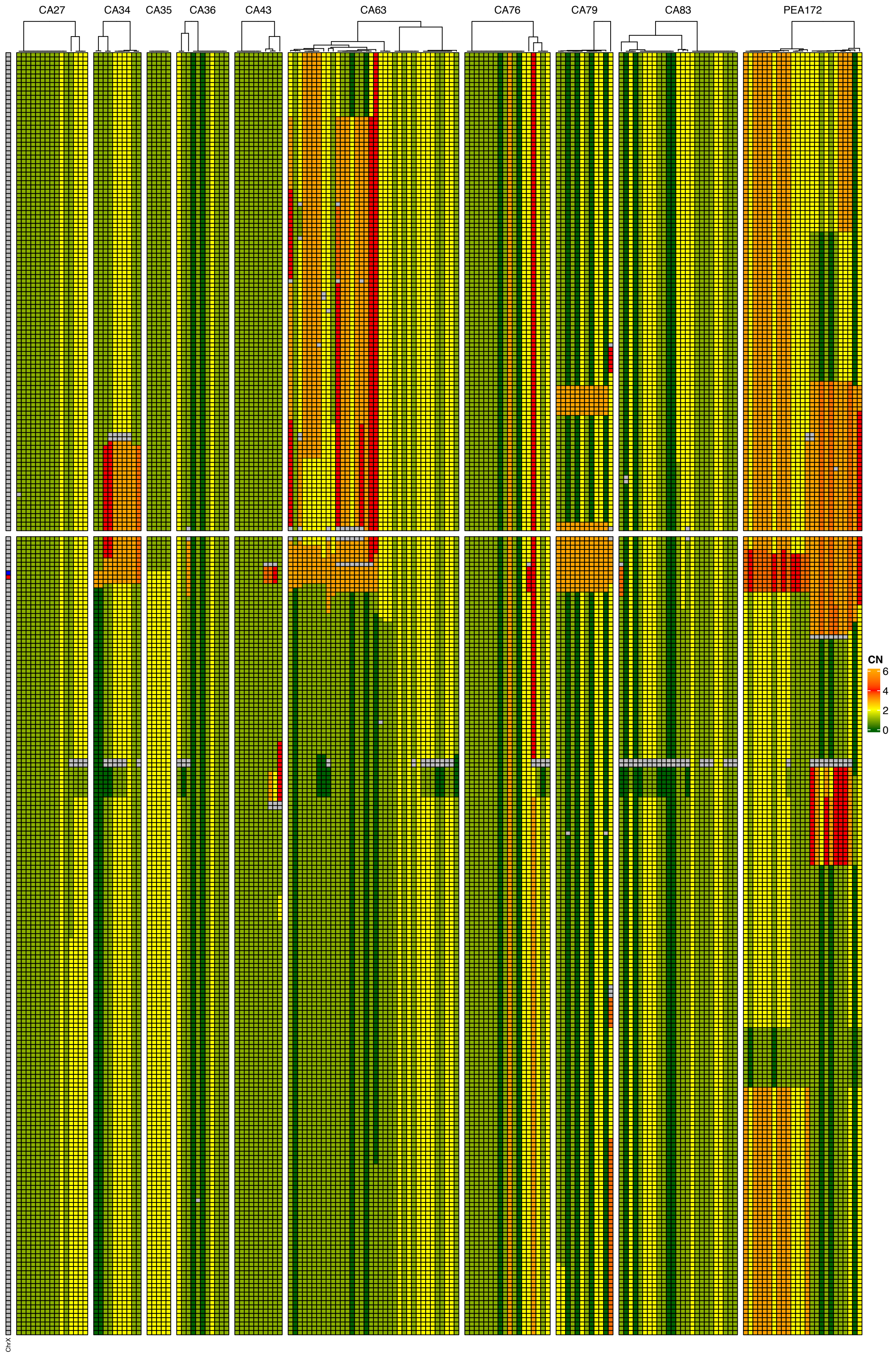
CA27
CA34
CA35
CA36
CA43
CA63
CA76
CA79
CA83
PEA172
CN
6
4
2
0
ChrX

33

Supplementary Figure 3. Copy number gain extends over a large area on chromosome X.

Heatmap showing the copy number of individual chromosome X bins (500 kb wide) for all 167 samples harvested post-mortem. Color scheme for the copy number shown in the legend. Chromosome X is shown on the left of the heatmap along with its bins covering *AR* gene (in red) and *AR*-associated centromeric enhancer (in blue). Metastases are ordered by patient identification as presented in **Figure 1** and within patients, based on hierarchical clustering (shown as dendrogram) of correlation distances calculated among copy number transitions on chromosome X.

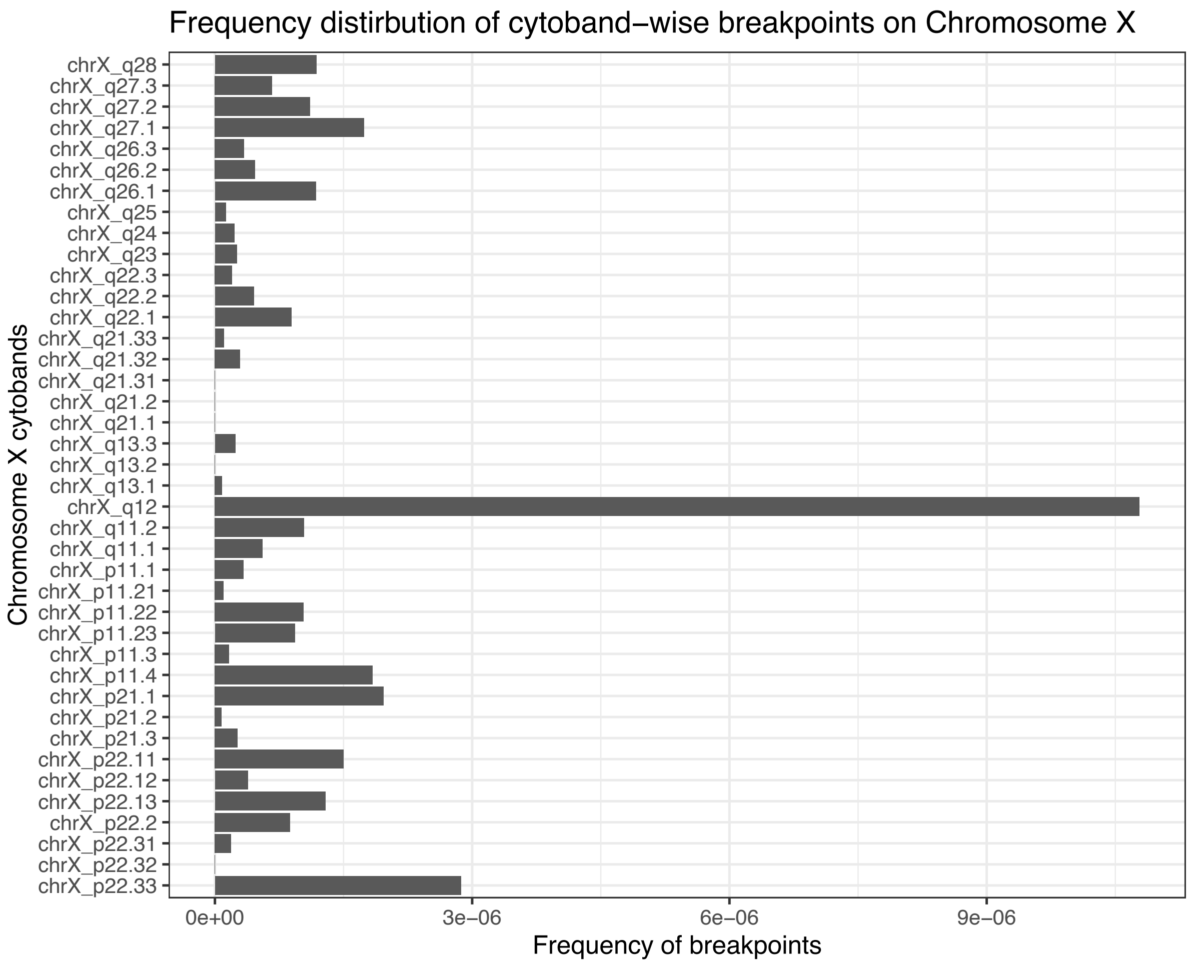

Supplementary Figure 4: Frequency distribution of breakpoints detected in chromosome X split by cytobands identifies the highest frequency at cytoband q12 (harboring *AR* gene). Frequency (in 22 tumors from 9 patients with high coverage WGS across chromosome X) represents the number of breakpoints in each cytoband normalized by the length (bp) of the respective cytoband.

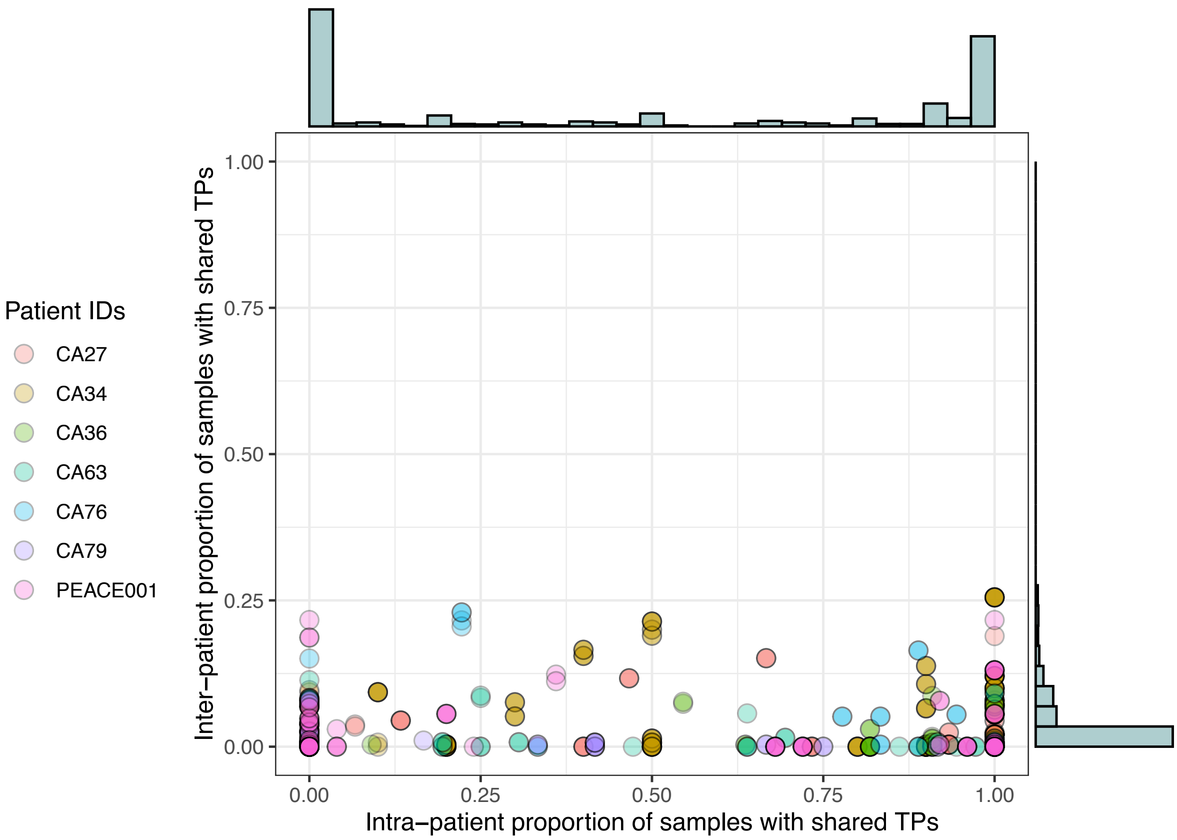

Supplementary Figure 5: Overlap of copy number transition points between post-mortem tumor samples and diagnostic biopsies within and across patients

Each dot represents the proportion of transition points (TPs) detected in a diagnostic biopsy sample (n=8 patients) that are shared with samples harvested post-mortem from the same patient (x-axis) or any metastases harvested from other patients (y-axis). Marginal histograms confirm that whilst occasional TPs are shared with metastases from other patients, all patients' diagnostic samples share a high proportion of TPs (common or truncal TPs) with metastases from the same patient.

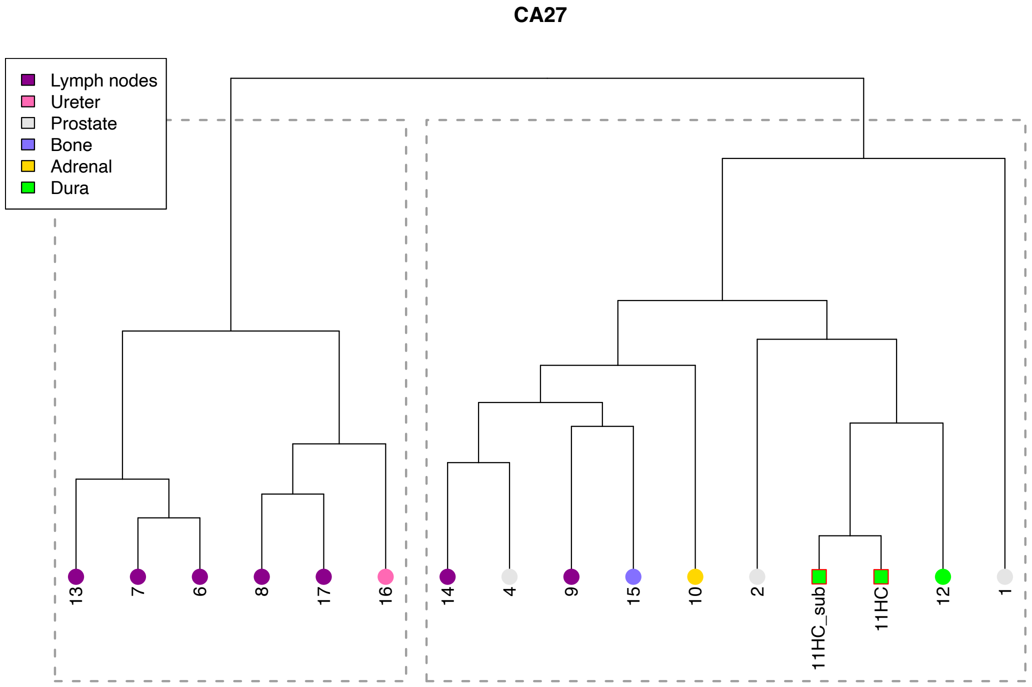
CA27
Lymph nodes
Ureter
Prostate
Bone
Adrenal
Dura
13
7
6
8
17
16
14
4
9
15
10
2
11HC_sub
11HC
12
1

64

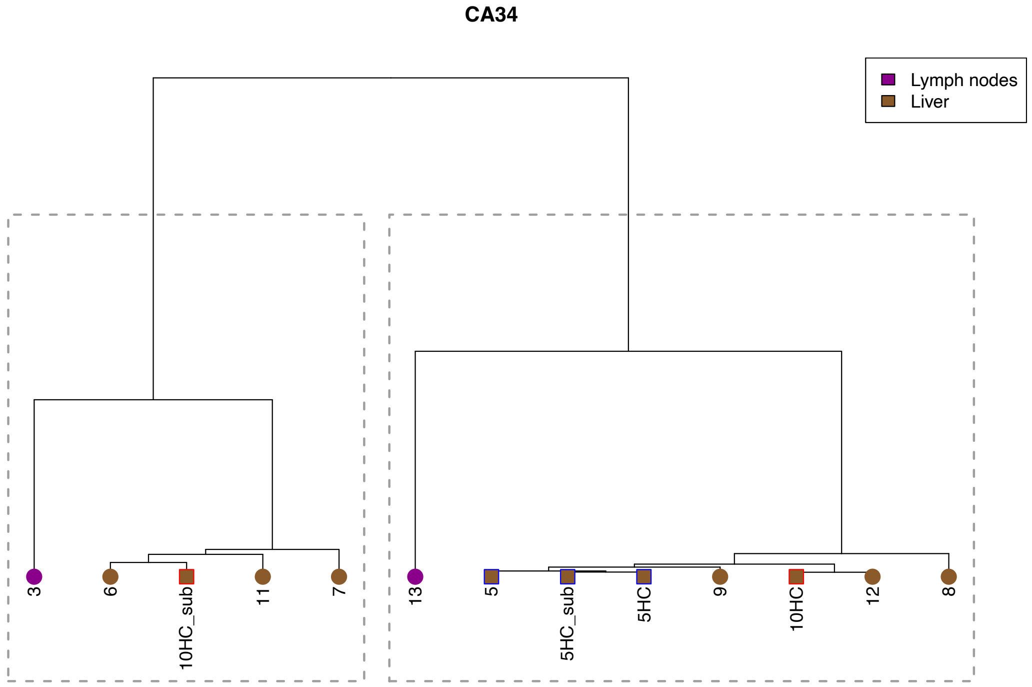
CA34
Lymph nodes
Liver
3
6
10HC_sub
11
7
13
5
5HC_sub
5HC
9
10HC
12
8

65

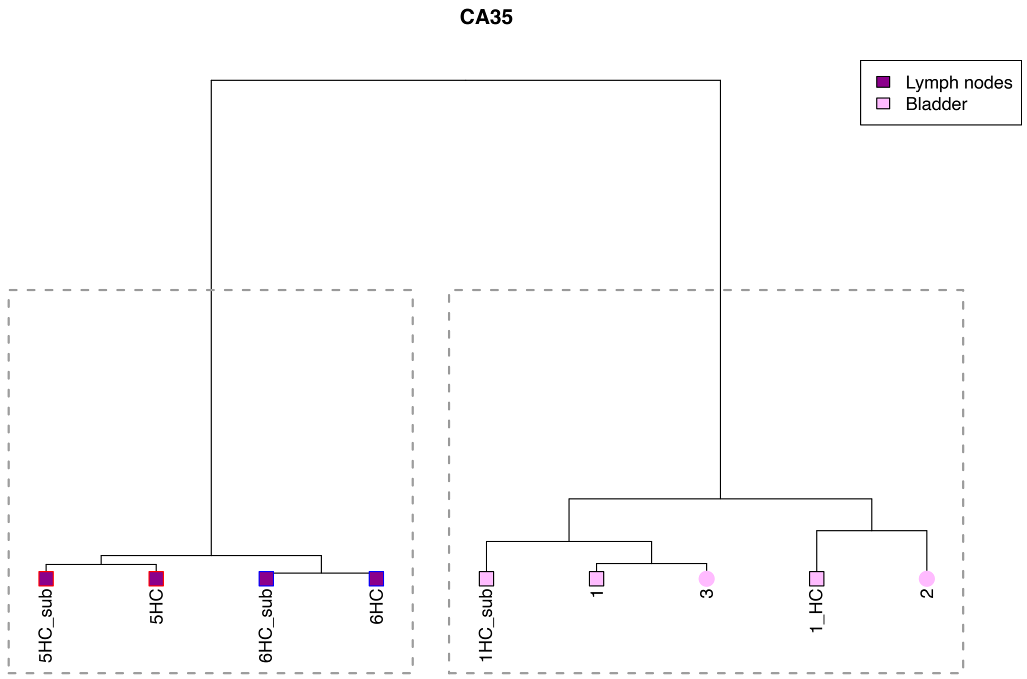
CA35
Lymph nodes
Bladder
5HC_sub
5HC
6HC_sub
6HC
1HC_sub
1
3
1_HC
2

66

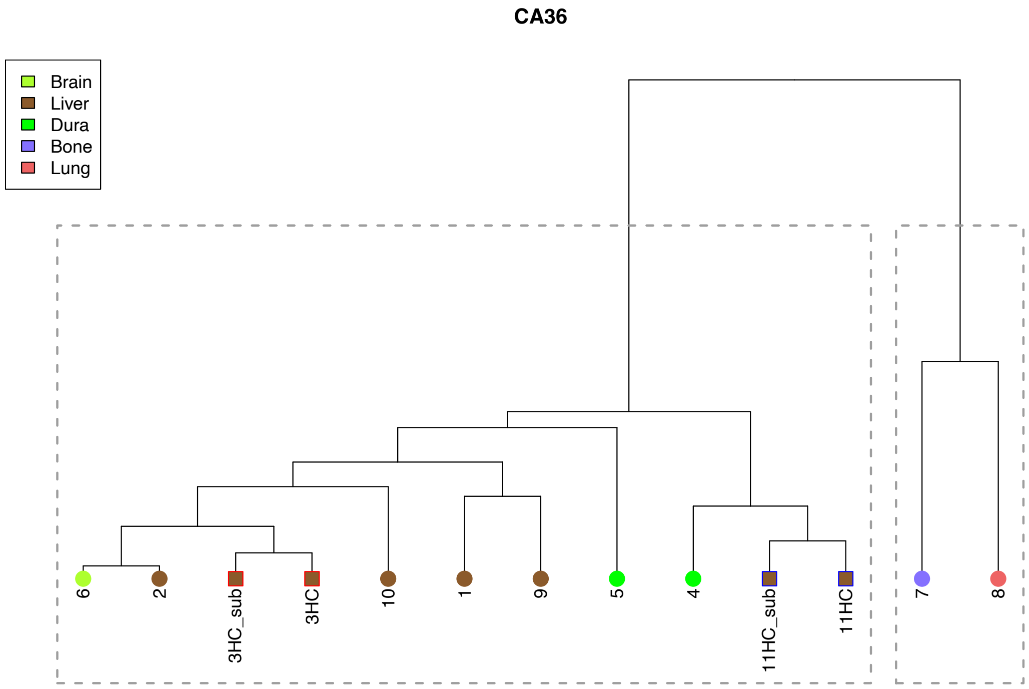
CA36
Brain
Liver
Dura
Bone
Lung
6
2
3HC_sub
3HC
10
1
9
5
4
11HC_sub
11HC
7
8

67

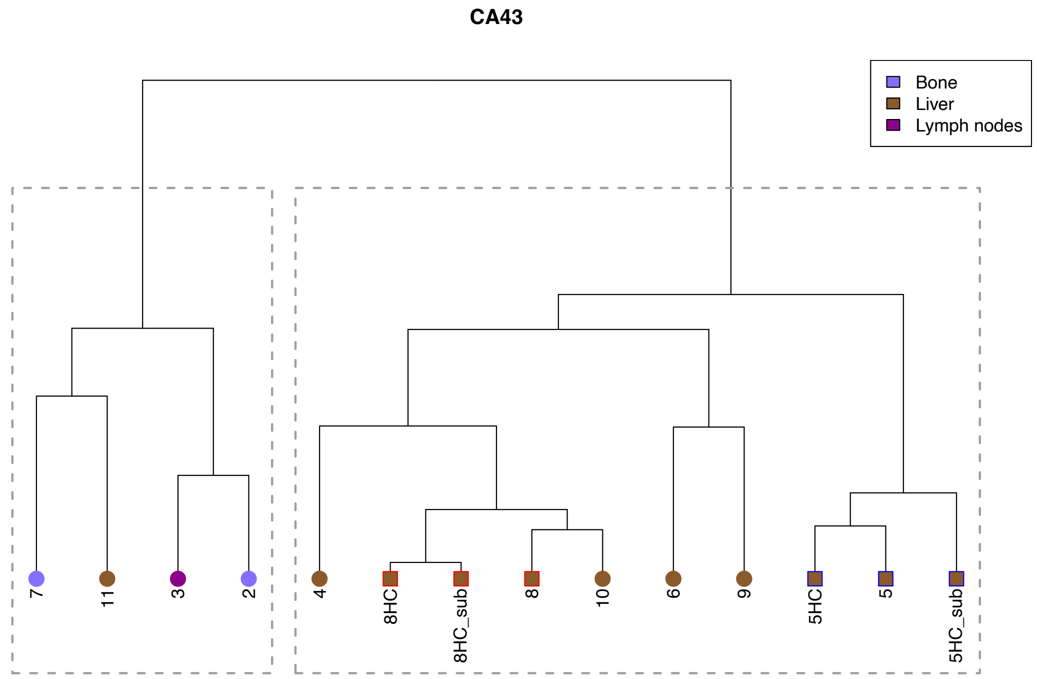
CA43
Bone
Liver
Lymph nodes
7
11
3
2
4
8HC
8HC_sub
8
10
6
9
5HC
5
5HC_sub

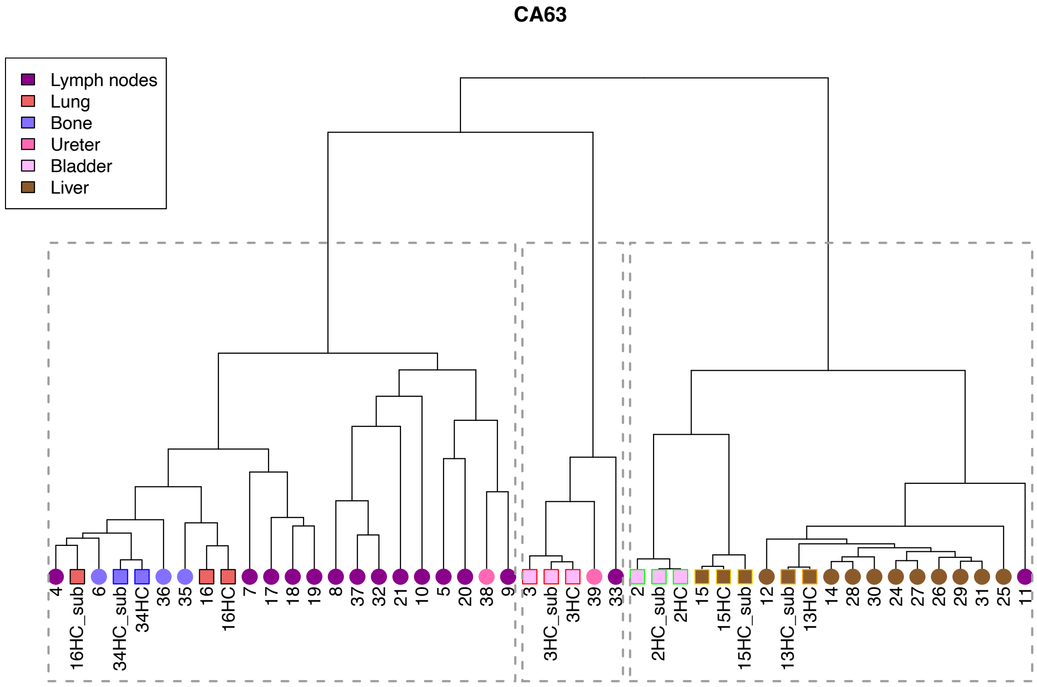
CA63
Lymph nodes
Lung
Bone
Ureter
Bladder
Liver
4
16HC_sub
6
34HC_sub
34HC
36
35
16
16HC
7
17
18
19
8
37
32
21
10
5
20
38
9
3
3HC_sub
3HC
39
33
2
2HC_sub
2HC
15
15HC
15HC_sub
12
13HC_sub
13HC
14
28
30
24
27
26
29
31
25
11

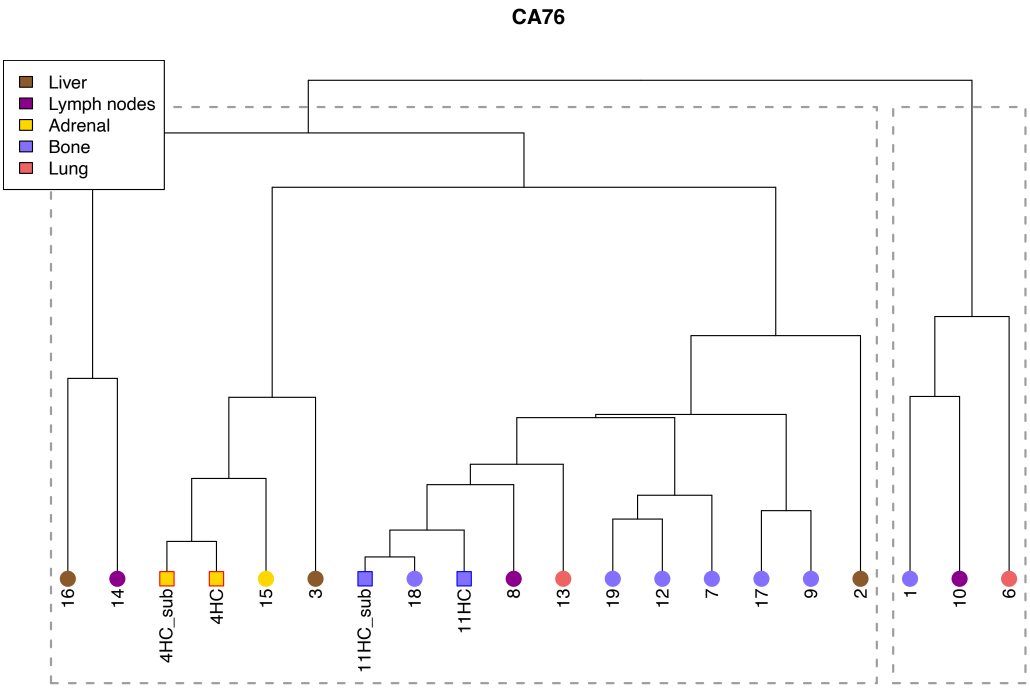
CA76
Liver
Lymph nodes
Adrenal
Bone
Lung
16
14
4HC_sub
4HC
15
3
11HC_sub
18
11HC
8
13
19
12
7
17
9
2
1
10
6

70

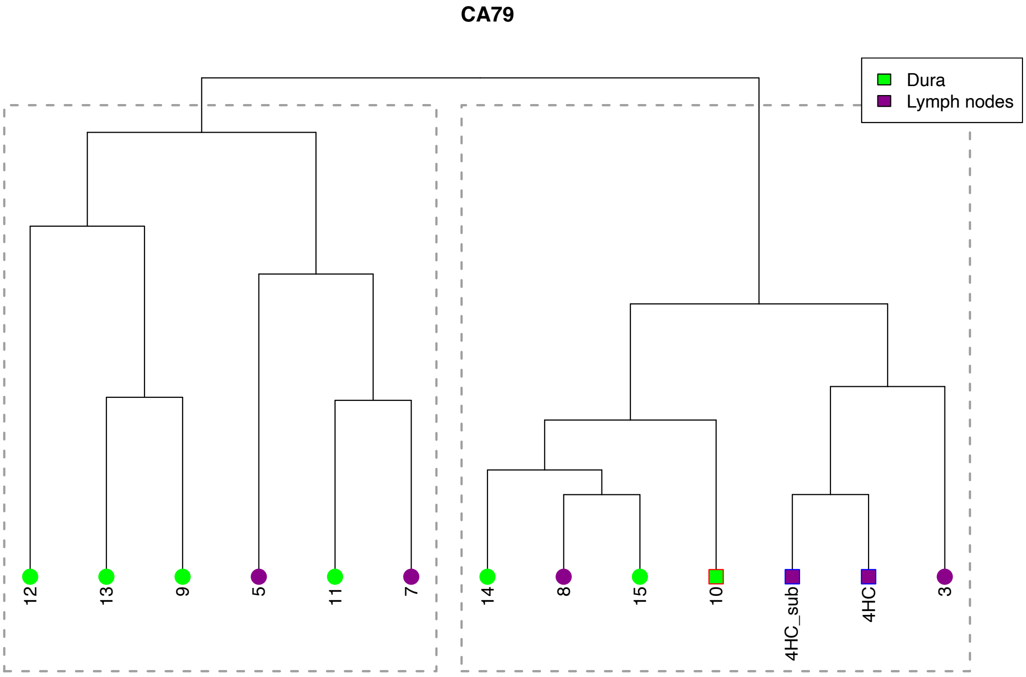
CA79
Dura
Lymph nodes
12
13
9
5
11
7
14
8
15
10
4HC_sub
4HC
3

71

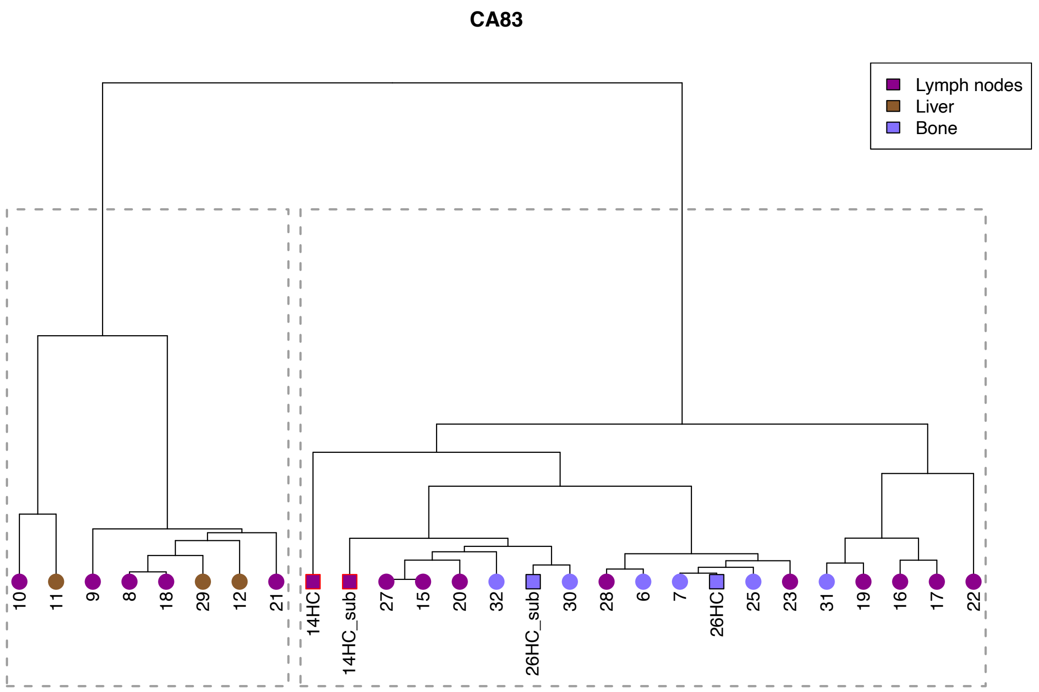

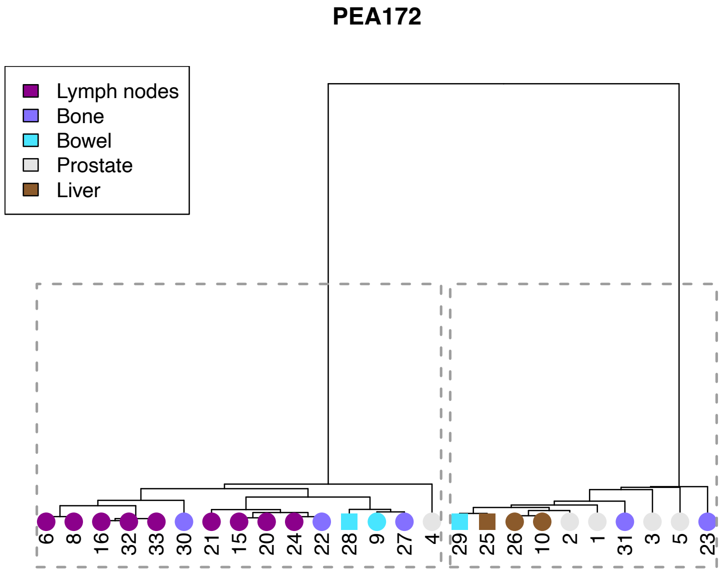

Supplementary Figure 6: Relationship assignment of metastases on a SCRATCH relational network

In SCRATCH relationship networks, tumors with high coverage sequencing ("HC") and/or subsampled versions of them ("HC_sub") were assigned predominantly close (or in the same cluster, denoted by grey dashed boxes) to the same samples with low coverage sequencing data. The organ sites are depicted with color-coded circles (color scheme in the legend) on

the terminal nodes and the samples in comparison (outlined with same-colored lines) are depicted in same-colored squares.

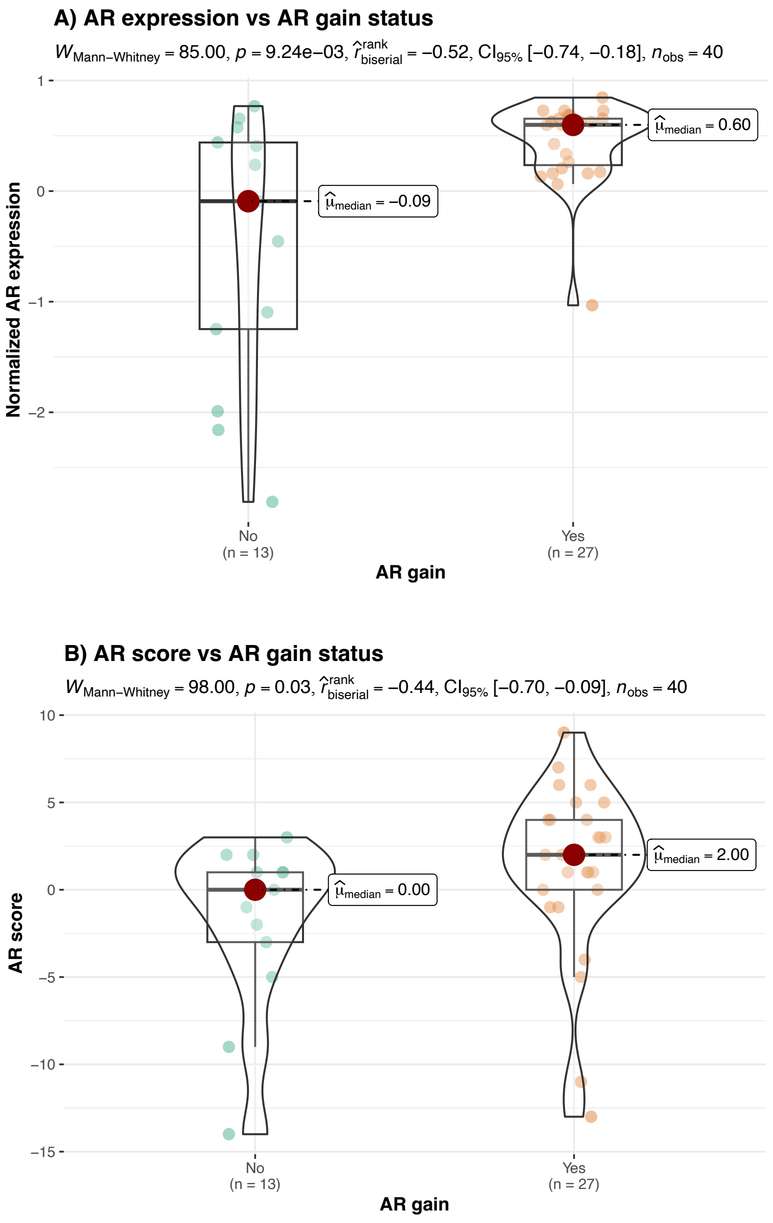

Supplementary Figure 7: Associations of AR expression and AR copy number with AR score for grouped tumors

A) Boxplot of normalized AR expression in metastases with *AR* copy number gain or not. B) Boxplots of the expression of AR-regulated genes (calculated as AR-score) in metastases with *AR* copy number gain (n=27 samples) or no gain (n=13 samples). The analysis was performed on AR-target gene sets described by Hieronymous and collaborators. Significance of the difference was calculated using Mann-Whitney (one-sided) test for both cases on RNA-

Seq data from patients CA63, CA76, CA83 and PEA172. The median value of each distribution is shown along the box's central horizontal line and the whiskers follow median ± 1.5*IQR pattern.

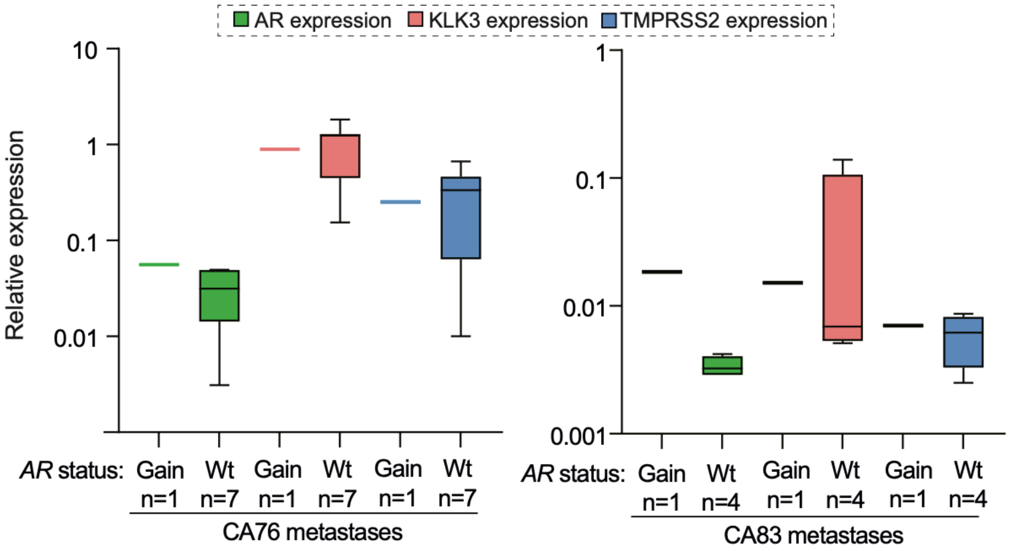

Supplementary Figure 8: Droplet digital PCR for AR, KLK3 and TMPRSS2 transcripts on individual tumor samples

Transcripts of AR and it's regulated genes *KLK3* and *TMPRSS2* measured by ddPCR are plotted on a logarithmic scale (y-axis) for patients CA76 and CA83, with tumors split by their *AR* copy number status (x-axis). In each box central line represents the mean and the whiskers represent mean ± IQR * 1.5 and p-values generated from the two-tailed Mann-Whitney U test are shown on the top for each comparing pair. The number of samples are shown below the x-axis – 8 samples (1 *AR* gain and 7 *AR* wild-type copy number) for CA76 and 5 samples (1 *AR* gain and 4 *AR* wild-type copy number) for CA83.

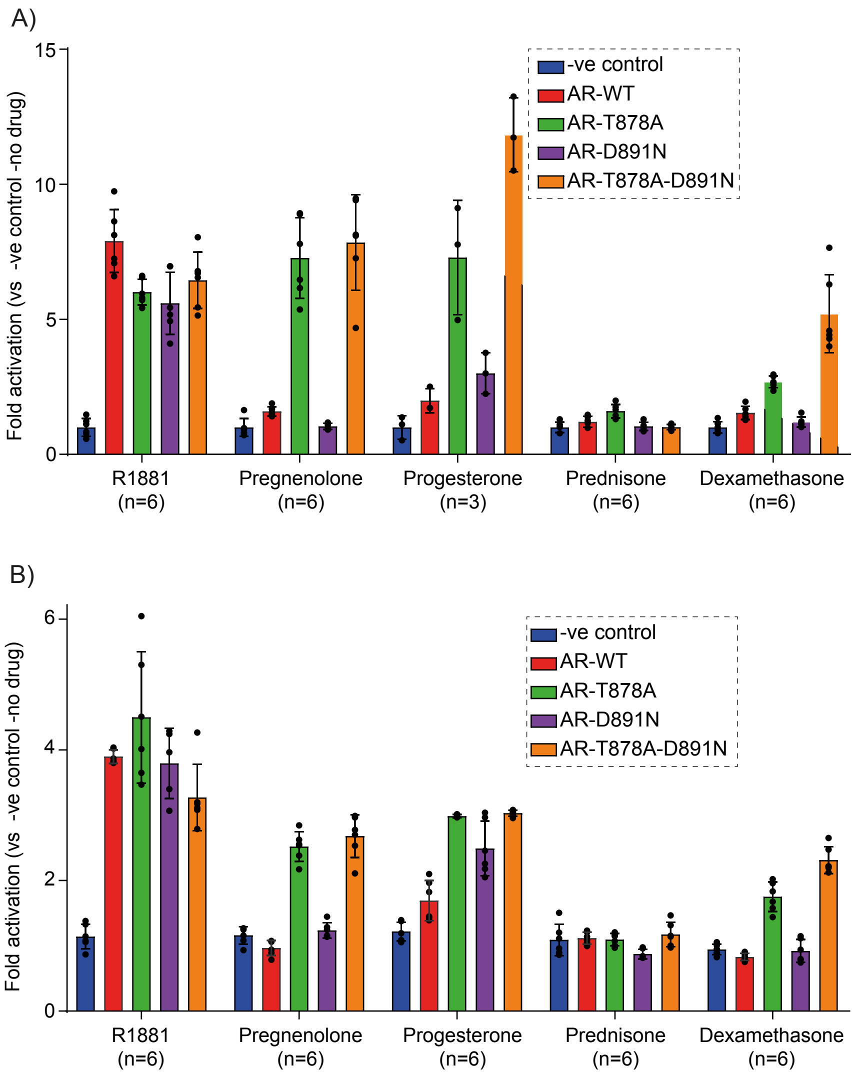

Supplementary Figure 9: Biological replicates of Reporter-luciferase assay reported in Figure 3f

Panel a) and b) shows two separate biological replicates of reporter-luciferase assay for the activation of wild-type and mutant AR (T878A and D891N, individually and combined) by clinically relevant ligands (R1881: Synthetic Androgen). 3 to 6 technical replicates (depicted by the 'n' numbers) were performed for each combination of ligand and *AR* mutation types (or wild-type).
