# Supplementary material for: Copy number architectures define treatment-mediated selection of lethal prostate cancer clones
Source: Nat Commun. 2023 Aug 10;14:4823. doi: 10.1038/s41467-023-40315-9 (PMC10415299; doi:10.1038/s41467-023-40315-9)
Supplement: Supplementary file 3 — Description of Additional Supplementary Files [file 41467_2023_40315_MOESM3_ESM.pdf]

Description of Additional Supplementary Files

File Name: Supplementary Data 1
Description: Clinical information of 10 patients.

File Name: Supplementary Data 2
Description: Detailed sample summary and coverage statistics.

File Name: Supplementary Data 3
Description: Allele frequency and copy number of AR pE710G and wild-type allele in CA43

File Name: Supplementary Data 4
Description: Copy numbers at autosomal Transition points

File Name: Supplementary Data 5
Description: Clonal somatic non-synonymous mutations

File Name: Supplementary Data 6
Description: Large areas of copy number gain detected in Chromosome X

File Name: Supplementary Data 7
Description: Inter- and intra-patient comparison of Structural variants

File Name: Supplementary Data 8
Description: Comparison of Transition Points

File Name: Supplementary Data 9
Description: AR expression and AR scores

File Name: Supplementary Data 10
Description: Custom capture panel design

File Name: Supplementary Data 11
Description: Sequence alignments of AR-V12 and proposed AR-V12 mimics

File Name: Supplementary Data 12
Description: TMPRSS2-ERG status
