# Supplementary material for: Copy number architectures define treatment-mediated selection of lethal prostate cancer clones
Source: Nat Commun. 2023 Aug 10;14:4823. doi: 10.1038/s41467-023-40315-9 (PMC10415299; doi:10.1038/s41467-023-40315-9)
Supplement: Supplementary file 16 — Reporting Summary [file 41467_2023_40315_MOESM16_ESM.pdf]

nature portfolio

Corresponding author(s): Gerhardt Attard

Last updated by author(s): Jul 7, 2023

Reporting Summary

Nature Portfolio wishes to improve the reproducibility of the work that we publish. This form provides structure for consistency and transparency in reporting. For further information on Nature Portfolio policies, see our Editorial Policies and the Editorial Policy Checklist.

Statistics

For all statistical analyses, confirm that the following items are present in the figure legend, table legend, main text, or Methods section.

n/a	Confirmed	
☐	☒	The exact sample size (*n*) for each experimental group/condition, given as a discrete number and unit of measurement
☐	☒	A statement on whether measurements were taken from distinct samples or whether the same sample was measured repeatedly
☐	☒	The statistical test(s) used AND whether they are one- or two-sided<br>*Only common tests should be described solely by name; describe more complex techniques in the Methods section.*
☐	☒	A description of all covariates tested
☒	☐	A description of any assumptions or corrections, such as tests of normality and adjustment for multiple comparisons
☐	☒	A full description of the statistical parameters including central tendency (e.g. means) or other basic estimates (e.g. regression coefficient) AND variation (e.g. standard deviation) or associated estimates of uncertainty (e.g. confidence intervals)
☐	☒	For null hypothesis testing, the test statistic (e.g. *F*, *t*, *r*) with confidence intervals, effect sizes, degrees of freedom and *P* value noted<br>*Give P values as exact values whenever suitable.*
☒	☐	For Bayesian analysis, information on the choice of priors and Markov chain Monte Carlo settings
☐	☒	For hierarchical and complex designs, identification of the appropriate level for tests and full reporting of outcomes
☒	☐	Estimates of effect sizes (e.g. Cohen's *d*, Pearson's *r*), indicating how they were calculated

Our web collection on statistics for biologists contains articles on many of the points above.

Software and code

Policy information about availability of computer code

Data collection	No software or code was used for data collection (See methods and supplementary method).
Data analysis	Mapping and data processing: BWA (v0.7.12), samtools (v1.3.1),picard-tools (v2.18.9), STAR (v2.7.10a_alpha_220517)<br>Somatic mutation calling and annotation: GATK4 Mutect2 and dNdScv (v-0.0.1.0)<br>Somatic structural variant calling: Delly (V-0.7.8)<br>Clonal Decomposition: Sclust (v1.1)<br>Statistical packages used for downstream analyses: R (v 4.2.2) and GraphPad Prism (academic license).<br>Few important software and packages for specific analyses:<br>Copy number variation analysis: R packages QDNAseq (v 1.34.0) and ACE (v 1.16.0) packages<br>For calculating tumor content: PLATYPUS v0.8.1.2 42, BEAGLE5 and SEQUENZA v2.1.2.<br>Relationship among metastatses: R package cluster (v2.1.4)<br>Congruence analysis: R package dendextend (v1.17.1)<br>Detection of gene fusion: Arriba (v2.1.0)<br>RNA-Seq count data and further normalization: HTSeq-count (v2.0.1) and R/Bioconductor package: Limma (v3.54.2)<br>Other R packages:<br>magrittr(v2.0.3)<br>dplyr(v1.1.1)<br>ggplot2(v3.4.1)<br>gridExtra(v2.3)<br>reshape2(v1.4.4)<br>tidyr(v1.3.0)

purrr(v1.0.1)
ggpubr(v0.6.0)
phylogram(v2.1.0)
For details see methods.
Also: https://github.com/AMMHasan/Copy-number-architectures-define-treatment-mediated-selection-of-lethal-prostate-cancer-clones.git

For manuscripts utilizing custom algorithms or software that are central to the research but not yet described in published literature, software must be made available to editors and reviewers. We strongly encourage code deposition in a community repository (e.g. GitHub). See the Nature Portfolio guidelines for submitting code & software for further information.

Data

Policy information about availability of data

All manuscripts must include a data availability statement. This statement should provide the following information, where applicable:

- Accession codes, unique identifiers, or web links for publicly available datasets
- A description of any restrictions on data availability
- For clinical datasets or third party data, please ensure that the statement adheres to our policy

The raw transcriptomic and genomic data generated in this study are available on request on the European Genome-phenome Archive under accession number EGAS00001006598 [https://ega-archive.org/studies/EGAS00001006598]. All researchers can obtain access by submitting a project proposal to the Data Access Committees (DAC) by contacting the corresponding author (G.A.). Requests will be handled within ~8 weeks. The DAC will also determine the length of permitted access dependent on the requirements of a specific project. Processed data (minimum dataset) generated in this study have been deposited in Zenodo database under DOI: 10.5281/zenodo.8125338 [https://zenodo.org/badge/latestdoi/651489188]58.

Human research participants

Policy information about studies involving human research participants and Sex and Gender in Research.

Reporting on sex and gender	Sex: Male. No further amendments of gender (compared to sex) reported by the patients.
Population characteristics	Ten patients involved in this study, nine have a median age 69 with a range of 56-72 years participating in CASCADE study and one with age of 65 years participating in PEACE study. Further details (if relevant) can me found in Supplementary table S1.
Recruitment	Patients (nine) were recruited in CASCADE community-based rapid autopsy study and (one) PEACE (NCT03004755) study.
Ethics oversight	CASCADE study: Human Research Ethics Committee of the Peter MacCallum Cancer Centre, Melbourne (HREC approval numbers: CASCADE 13/122).<br>PEACE study: London (Dulwich) Human Research Ethics Committee (13/LO/0972)

Note that full information on the approval of the study protocol must also be provided in the manuscript.

Field-specific reporting

Please select the one below that is the best fit for your research. If you are not sure, read the appropriate sections before making your selection.

☒ Life sciences ☐ Behavioural & social sciences ☐ Ecological, evolutionary & environmental sciences

For a reference copy of the document with all sections, see nature.com/documents/nr-reporting-summary-flat.pdf

Life sciences study design

All studies must disclose on these points even when the disclosure is negative.

Sample size	201 fresh frozen tumor samples from 11 different organs.<br>Single plasma samples from eight men at death.<br>33 archived formalin-fixed paraffin embedded (FFPE) tumor samples.<br>The inclusion criterion for this analysis was that the patient died from metastatic castration-resistant prostate cancer (mCRPC). All cases analyzed were included in this report. The number of samples to be included in the analysis was not pre-defined based on power calculations and we aimed to include the maximum number with sufficient tumor content. Tumor samples were obtained post-mortem, and site of biopsy was carefully annotated and photographed. Where possible, formalin-fixed paraffin-embedded (FFPE) tumor samples acquired whilst the patient was alive were retrieved. There was no randomization or blinding involved.
Data exclusions	We exclude samples with a tumor fraction of less than 0.2 (not including), namely 34 fresh-frozen post-mortem, three plasma and nine formalin-fixed archival samples.
Replication	Figure 3f and Supplementary Figure 9 A and B include 3 panels where each panel is a unique biological replicate of the same experiment. Within each biological replicate we included 3-6 technical replicates.

Randomization	No randomization involved as not relevant given the pragmatic approach of harvesting postmortem tumor samples in a cohort size of only 10 patients.
Blinding	No blinding involvedas not relevant given the pragmatic approach of harvesting postmortem tumor samples in a cohort size of only 10 patients.

Reporting for specific materials, systems and methods

We require information from authors about some types of materials, experimental systems and methods used in many studies. Here, indicate whether each material, system or method listed is relevant to your study. If you are not sure if a list item applies to your research, read the appropriate section before selecting a response.

Materials & experimental systems

n/a	Involved in the study
☒	☐ Antibodies
☐	☒ Eukaryotic cell lines
☒	☐ Palaeontology and archaeology
☒	☐ Animals and other organisms
☐	☒ Clinical data
☒	☐ Dual use research of concern

Methods

n/a	Involved in the study
☒	☐ ChIP-seq
☒	☐ Flow cytometry
☒	☐ MRI-based neuroimaging

Eukaryotic cell lines

Policy information about cell lines and Sex and Gender in Research

Cell line source(s)	American Type Culture Collection (Cat #CRL-1435)
Authentication	Cells from PC3 cell-line were routinely checked for Mycoplasma and verified by fingerprinting (HPA cultures or Eurofins)
Mycoplasma contamination	Tested negative for Mycoplasma.
Commonly misidentified lines (See ICLAC register)	None

Clinical data

Policy information about clinical studies

All manuscripts should comply with the ICMJE guidelines for publication of clinical research and a completed CONSORT checklist must be included with all submissions.

Clinical trial registration	REC number 13/LO/0972
Study protocol	Can be provided in the supplementary section
Data collection	CASCADE: Peter MacCallum Cancer Centre, Melbourne, Australia (span: between October 2013 and August 2017)<br>PEACE: University College Hospitals, UK (span: between June 2019 and to date)
Outcomes	Relationship of tumor at death determined by SCRATCH algorithm and the this relationship was further assessed by the colinearity of node distance and common clonal non-silent mutations. (Details in the result section)
